# Talent Flow Network, the Life Cycle of Firms, and Their Innovations

**DOI:** 10.3389/fpsyg.2022.788515

**Published:** 2022-05-20

**Authors:** Bo Sun, Ao Ruan, Biyu Peng, Wenzhu Lu

**Affiliations:** ^1^School of Economics and Management, South China Normal University, Guangzhou, China; ^2^International Business School, South China Normal University, Guangzhou, China; ^3^School of Business Administration, South China University of Technology, Guangzhou, China

**Keywords:** talent flow network, firm life cycle, network embedding breadth, network embedding depth, innovations, resume data

## Abstract

This paper explores how talent flow network and the firm life cycle affect the innovative performances of firms. We first established an interorganizational talent flow network with the occupational mobility data available from the public resumes on LinkedIn China. Thereafter, this information was combined with the financial data of China’s listed companies to develop a unique dataset for the time period between 2000 and 2015. The empirical results indicate the following: (1) The breadth and depth of firms’ embedding in the talent flow network positively impact their innovative performances; (2) Younger firms’ innovations are mostly promoted by the breadth of network embedding, but this positive effect weakens as firms increase in age; (3) Mature firms’ innovations are primarily driven by the depth of network embedding, and this positive effect strengthens as firms increase in age. This paper enriches and deepens the studies of talent flow networks, and it provides practical implications for innovation management based on talent flow for various types of firms at different development stages.

## Introduction

The existing literature acknowledges the significance of innovation in improving the performances of firms and maintaining their long-term competitive advantages (Clark and Guy, 1998; Mone et al., 1998). However, with the increasing complexity of the innovation process, it has become quite challenging for individual organizations to continuously innovate with limited information, knowledge, and technology. Hence, firms must expand beyond their organizational boundaries and use external resources to foster further innovations (Chesbrough, 2003). The Research and Development (R&D) of COVID-19 vaccines illustrates the significance of broadening organizational boundaries to acquire external resources. Since the beginning of the epidemic, significant attention was focused on the development of highly effective and safe vaccines (Vuong et al., 2022). Considering previous experience, it may have taken 10–15 years to develop a new vaccine if medical institutions worked on an individual basis (Wherry et al., 2021). However, the COVID-19 vaccine was successfully developed within a year by using interorganizational cooperation and resource sharing (Vuong et al., 2022).

A talent flow network is a firm-level social network that is shaped by the movement of talent across organizations (Dokko and Rosenkopf, 2010). In this type of network, the nodes represent individual firms and the connecting lines between the nodes represent the movement of talent among firms. Compared to other types of networks, such as alliance networks (Weng et al., 2014) and collaboration networks (Wang et al., 2014; Su et al., 2021), talent flow networks have an obvious spillover effect that allows firms to obtain important innovative resources from other organizations more directly and deeply (Song and Wu, 2003; Shipilov et al., 2017). Several existing studies have found that firms that are in advantageous positions in a talent flow network acquire more abundant and high-quality innovative resources that consist of information, knowledge, and technology. This acquisition of resources influences the innovative behavior and performance of firms (Dokko and Rosenkopf, 2010; Godart et al., 2014; Shipilov et al., 2017). Although some studies have examined the nature, importance, and impact of the talent flow network (Dokko and Rosenkopf, 2010; Godart et al., 2014; Shipilov et al., 2017), most of the existing literature focuses on the embedding breadth of the network and its heterogeneous resources, while overlooking the embedding depth of the network, which may lead to more pivotal innovative resources.

In fact, not all firms prioritize the acquisition of heterogeneous, innovative resources. Coad et al. (2016) as well as Akcigit and Kerr (2018) explain that firms usually adjust their innovative strategies at different stages of development. Younger firms tend to adopt exploratory innovative strategies that are more radical to seize new markets. However, mature firms that have a stable source of income are more likely to use exploitative innovation to refine and optimize their existing products. Thus, younger firms usually obtain more diversified innovative resources from the talent flow network to produce more innovative products, but mature firms search more deeply in the network for higher-level resources that optimize and improve their existing products. Consideration of these concepts leads to an important question. For firms at different life stages, what network embedding strategies are suitable and effective for enhancing their innovative performances?

This paper attempts to address this question by exploring the dynamic impact of the breadth and depth of network embedding on the innovation of firms at different stages. We conducted tests with a unique resume dataset obtained from LinkedIn (China). Specifically, we introduce two concepts in our paper that firms may use as strategies in the talent flow network—the breadth and the depth of the external resource search (Laursen and Salter, 2006). Network embedding breadth measures the diversification of firms’ connections with the outside world. When firms have a greater embedding breadth, they are connected to other organizations through talent flow in a more comprehensive manner, and they can access innovative resources that are more diversified. On the other hand, network embedding depth indicates how closely and strongly firms are connected to the leading organizations in the network. Greater depth implies that firms have stronger connections with the leading companies in their field and can acquire innovative resources that are more specialized. Thus, this study will explore two questions. First, do network embedding breadth and depth improve the innovation performances of firms? Second, since the impact of network embedding breadth and depth on innovation changes as firms increase in age, does network embedding breadth or depth benefit younger enterprises or mature firms?

We took several steps to obtain the unique dataset for our study. First, we collected resumes from the public internet space (i.e., LinkedIn China) and identified the top managers and technology talent by using position titles. Second, we developed the talent flow network based on the work experiences of the subjects. Next, the structure indexes were calculated to measure network embedding breadth and depth. Finally, we matched firms to the corresponding data from the China Stock Market and Accounting Research (CSMAR) Database and obtained a dataset that contained 3,027 samples from the time period of 2000–2015. Our analysis of the dataset indicated the following empirical results: (1) Both network embedding breadth and depth significantly improve firms’ innovative performances; (2) Young firms’ innovations benefit more from network embedding breadth, but this positive effect decreases as firms increase in age; (3) Mature firms’ innovations are mainly enhanced by network embedding depth, and this impact strengthens as firms increase in age. Additional analysis indicates that these results are still applicable to non-high-tech companies, SOEs (State-owned enterprises), and non-SOEs. Furthermore, high-tech companies’ innovations are mainly driven by the embedding breadth.

Considering the existing studies in this area, this paper has two main contributions. First, by using the perspectives of network embedding breadth and depth to systematically examine the impact of firms’ network embedding features on their innovative performances, this paper supplements the previous studies that focus on this impact solely from a network centrality perspective. In addition, this paper enriches the literature regarding the relationship between the talent flow network and the innovations of firms. Second, by introducing the concept of the enterprise life cycle into the study, this paper investigates how network embedding breadth and depth have a dynamic relationship with the innovations of firms who are in different development stages. This work not only enriches the studies of the relationship between the talent flow network and the innovations of firms, but also provides practical implications for firms to maximize the positive effects of the talent flow network on their innovations.

We have organized this paper to explain the relevant theories and literature and to develop the hypotheses in section “Theories and Hypotheses.” Thereafter, section “Materials and Methods” presents the research design, including the selection of variables and the development of empirical models. Section “Results” provides the empirical results analysis, the robustness checks, and the additional analysis, and section “Discussion” concludes the paper with our discussion of the study.

## Theories and Hypotheses

### Enterprise Innovation

Research on enterprise innovation can be traced back to Schumpeter’s theory of innovation and creative destruction. Schumpeter explained that innovation is a process of integrating resources to create new values, which is crucial to both enterprise development and national economic growth (Schumpeter, 1942). Thereafter, scholars from many fields, such as economics, strategic management, organizational behavior, and psychology, conducted various studies of innovation. Innovation has developed into a multidimensional concept that refers to individual creativity, organizational innovation, and other characteristics (Vuong and Napier, 2014). Accordingly, this paper focuses on innovation at the enterprise level and specifically, innovation used to develop new products or new ideas (Damanpour and Gopalakrishnan, 2001). This paper generally refers to firms’ new products, services, or business patterns that are enhanced by the processing and transformation of innovative resources (Baldwin and Gellatly, 2003).

Many previous studies suggest that internal and external resources and factors affect the innovative performances of firms. According to these studies, internal factors, such as organizational structure, strategy, knowledge learning, size and management characteristics, significantly affect firms’ innovations (Cohen and Levinthal, 1989; Damanpour, 1991, 1996; Fagerberg et al., 2005). The previous studies also explain that external factors, such as the market structure, the industry characteristics, and the competitive environment, affect the innovations of firms (Cohen and Levinthal, 1989; Balachandra and Friar, 1997; Tidd, 2001; Flor and Oltra, 2004).

However, an increasing number of studies have explained that the innovation of modern enterprises relies more on interorganizational exchanges and cooperation (Landry et al., 2002). Because of the intersection between social network theory and innovation studies, the exploration of the innovations of firms from the social network perspective has become a new trend that provides an important theoretical angle. Research indicates that by using external social networks, firms obtain abundant resources, such as information, knowledge, and technology, that contributes to additional innovations (Dokko and Rosenkopf, 2010; Godart et al., 2014; Shipilov et al., 2017). In this process, social networks, such as cooperation networks and alliance networks, significantly impact firms’ innovative performances (Wang et al., 2014; Weng et al., 2014; Su et al., 2021).

Collectively, the existing literature has investigated the development of firms’ innovations from the perspectives of resource composition and resource processing. First, from the resource composition perspective, researchers believe that the key to fostering innovation is to possess and control innovative resources. Hence, researchers focus on what resources determine firms’ innovations and how firms acquire these unique and valuable resources, such as information (Vuong and Napier, 2014), knowledge (Nonaka, 1991), technology, and talent (Godart et al., 2014). Second, from the resource processing perspective, researchers believe that the combination and transformation of resources has a crucial influence on the formation of innovations, and therefore, these researchers focus on the formation of innovations within firms. For example, Vuong and Napier (2014, p. 304) present an innovation formation model based on information processing and explain that “out of discipline thinking,” “within discipline expertise,” and a “disciplined process” are the key factors in achieving innovations.

Considering the above researches, we chose to conduct our study from a resource composition and acquisition perspective. Therefore, this paper mainly explores the resources that firms acquire using different embedding strategies in the talent flow network and the effect of these heterogeneous resources on the formation of innovations.

### Network Embedding Breadth and Network Embedding Depth

The breadth and depth of the external resource search are two important dimensions of the search for innovative resources for firms (Laursen and Salter, 2006). The breadth of the search refers to the number of external resources. A greater breadth provides more heterogeneous resources for firms. The depth of the search measures how deeply firms explore the network for critical resources. A greater depth helps firms obtain pivotal and specialized resources from the network (Katila and Ahuja, 2002; Laursen and Salter, 2006).

The talent flow provides an effective channel for firms to acquire external resources, including information, knowledge, and technology. Research shows that firms not only acquire resources from external organizations through the talent flow process, but also gain resources through the backflow talent process that allows firms to maintain connections with former employees and observe the developing trends of their new employers (Shipilov et al., 2017). Therefore, by purposefully managing the talent flow, firms establish effective connections with specific external organizations, and they seek and acquire critical innovative resources via the resource spillover effect in the talent flow network.

Based on the concepts of external resource search breadth and depth (Laursen and Salter, 2006), our study suggests that firms usually apply two embedding strategies when using the talent flow network—network embedding breadth or network embedding depth. The breadth measures the diversity of firms’ connections with external organizations. Greater breadth means more diversification among the connected organizations in terms of their types (Shipilov et al., 2017; Wang et al., 2017), locations (Bell and Zaheer, 2007), and technology background (Lahiri and Narayanan, 2013). This variety provides useful information, knowledge, and technology for firms. The depth estimates how closely firms are connected with the key organizations in the network. Greater depth means stronger connections with the key enterprises in the field, such as competitors and research institutes within the same industry. Also, the intensified connections may provide additional specialized information, knowledge, and technology for firms (Katila and Ahuja, 2002; Laursen and Salter, 2006).

### Network Embedding Breadth and Firms’ Innovation Performance

Network embedding breadth is crucial to the innovative performances of firms for several reasons. According to the resource-based view, human capital consisting of talented individuals and their knowledge and skills are pivotal for firms to gain competitive advantages (Wernerfelt, 1984; Wright et al., 1994). Human capital significantly impacts the innovation activities of firms (Ployhart et al., 2014). This paper assumes that the broader the firms become embedded in the talent flow network, the more information, knowledge, and technology the firms gain from the resource spillover effect of the talent flow. These various resources are useful for firms to identify innovation opportunities and enhance R&D and innovation capabilities, which improves their innovation outcomes.

First, network embedding breadth provides diversified resources that help to identify promising innovative opportunities. This reduces innovation risks and improves the success rate of innovation activities. Long development cycles, high investment, and difficult transformation usually characterize the R&D process for innovative products (Green et al., 1995). Therefore, identifying a product development strategy that provides good market potential and high technical viability reduces the innovation risk and promotes the successful marketing of new products in the early stages in the life of the company (Schoonhoven et al., 1990). Greater network embedding breadth enables firms to acquire customer demand information from different regions and comprehend the pioneering technical issues and development trends of different industries (Lingo and Omahony, 2010; Shipilov et al., 2017; Wang et al., 2017). This allows firms to quickly recognize appropriate innovation opportunities with good market prospects and achieve better innovation performances.

Second, introducing more diversified resources by using the talent flow network enhances firms’ innovation performances. From the perspective of knowledge search and reorganization, innovation is a novel combination of different knowledge elements. The scope of the knowledge search and the method of reorganization determine firms’ innovative capacities (Savino et al., 2017). Kim and Kogut (1996) have explained that the potential of knowledge elements can be drained if they are repetitively utilized, and this hinders additional innovation. When firms introduce new knowledge or new combinations of knowledge elements, they enhance their potential for knowledge reorganization and innovation (Katila and Ahuja, 2002). Greater embedding breadth in the talent flow network allows firms to obtain various kinds of knowledge and technology (Lingo and Omahony, 2010; Wang et al., 2017). When firms reorganize these newly acquired resources with existing knowledge elements, they achieve greater innovation outcomes (West and Bogers, 2014). The previous empirical research has already established this concept—a broader talent source leads to better innovation outcomes (Godart et al., 2014; Shipilov et al., 2017).

Considering these principles, we posit our first hypothesis:

**Hypothesis 1: Greater network embedding breadth enhances the innovation performance of firms.**

### Network Embedding Depth and Firms’ Innovation Performance

Network embedding depth is important to the innovative outcomes of firms for several reasons. This paper suggests that network embedding depth is important to firms innovative outcomes. When firms are deeply embedded in the talent flow network, they acquire more specialized information, knowledge, and technology because of the spillover effect of the talent flow. These specialized resources help firms optimize the work process for innovative products, develop new products by refining existing products, and enhance their innovation performances.

First, increasing the networking embedding depth enables firms to frequently seek and acquire advanced professional knowledge and technology from peer organizations (Laursen and Salter, 2006). This deepens the comprehension of firms of their own knowledge and technology and allows them to develop new combinations of the available knowledge elements (Katila and Ahuja, 2002; Laursen and Salter, 2006). Thus, firms may develop new paradigms or patterns of knowledge utilization (Macher and Boerner, 2012). New combinations of knowledge elements help perfect existing innovations and develop new innovations (Grant, 1996). In addition, new patterns of knowledge utilization promote problem-solving efficiency and optimize the process for developing innovative products (Eisenhardt and Tabrizi, 1995). These activities enhance firms’ overall innovative performances.

Second, greater depth allows firms to seek and obtain more cutting-edge professional resources from the research institutes in the field. This resources help firms to upgrade their available knowledge systems and technologies and improve their comprehension of their professional resources. Greater depth also helps firms integrate and utilize both old and new knowledge elements and technologies, so they can develop new technologies and more innovative products (Laursen and Salter, 2006; West and Bogers, 2014).

These principles lead to the second hypothesis for this paper.

**Hypothesis 2: Greater network embedding depth leads to better innovation performance for firms.**

### Talent Flow Network, the Life Cycle of Firms, and Their Innovation Performance

Like living organisms, firms experience several life cycles, including the start-up, growth, maturation, and regression stages (Van Wissen, 2002). At different stages, diverse market, economic, and technological issues confront firms. Firms must adopt various strategies to survive and enhance their performances as much as possible (Agarwal and Gort, 2002). As the Schumpeterian growth model explained, firms change the novelty of their innovations at different stages. Some empirical studies have also explained that as the age of the firm increases, both the inputs and outputs of innovation decline (Huergo and Jaumandreu, 2004; Balasubramanian and Lee, 2008). Therefore, to maximize innovation outcomes, firms must adjust their innovation strategies to correlate with their development stages.

This paper postulates that when considering innovative performances, younger firms benefit more from the diversified resources of network embedding breadth, but mature firms gain more from the specialized resources of network embedding depth. This is true because the market objectives and innovation features of firms vary at different stages.

From the perspective of market objectives, young firms often lack market visibility and breakthrough products when they enter a new market. Thus, the most important market objective for these firms is to gain market recognition by introducing novel products rather than to make profits (Klette and Skrotum, 2004; Coad et al., 2016). On the other hand, older companies usually have mature, innovative products in the market and stable sources of income. For these companies, it is more important to optimize their existing products, satisfy their customer demand, and decrease production costs. By doing so, these companies maximize the profit from their innovative products and recover their R&D expenditures (Coad et al., 2016).

Greater network embedding breadth allows younger firms to acquire more abundant and diversified information, knowledge, and technology because of the spillover effects of the talent flow process (Godart et al., 2014; Shipilov et al., 2017). These various resources help firms identify the dynamic market demand and recognize the unique innovation opportunities in the market (Narver and Slater, 1990; Schweisfurth, 2017). Additionally, the heterogeneous knowledge and technology resources allow these younger firms to explore more original concepts and develop more innovative products (Oerlemans et al., 2013; Jung and Lee, 2016).

Considering this information, we posit the third hypotheses for this paper.

**Hypothesis 3: The innovation of young firms benefits more from the diversified resources of network embedding breadth, but this positive impact declines as firms increase in age.**

New firms have relatively flexible innovation patterns. At the time of start-up, new firms are still developing their knowledge and technology systems, and they have not fully developed their management systems and cooperative cultures. Thus, new firms generally have a wide latitude in the innovation process (Petruzzelli et al., 2018; Ardito et al., 2021). In contrast, mature firms have engaged in years of exploration and accumulation, and have already established stable knowledge, technology systems, and product development patterns. This helps mature firms identify and utilize specific knowledge and technologies more efficiently.

Because mature firms have already stabilized their management systems and cooperative cultures, they may be hindered in recognizing and utilizing heterogeneous innovative resources in the talent flow network (Ardito et al., 2021). This occurs when mature firms attempt to make exploratory innovations with these resources, and they forego their stable income from existing innovative products and their established knowledge and technology systems (Henderson and Clark, 1990). Mature firms should identify and use these new resources to rebuild their knowledge and technology systems (Criscuolo et al., 2012), but this transitional activity may lead to high friction costs. Consequently, mature firms are better suited for exploitative innovations that focus on the refinement and improvement of their existing products.

When older firms become more deeply embedded in the talent flow network, they obtain professional and pioneering information, knowledge, and technology through the spillover effects of the talent flow (Katila and Ahuja, 2002; Laursen and Salter, 2006). This acquisition of resources allows firms to grasp the micro changes in their customer and market demand, which helps firms refine their existing products and satisfy their market demand more effectively (Yang and Li, 2011). Additionally, the introduction of new resources may improve the available knowledge and technology systems, shape more sophisticated innovative technologies, and establish technological barriers. These activities help strengthen firms’ competitive advantages in the market (Lopez-Vega et al., 2016).

These principles lead to the fourth hypothesis of this paper.

**Hypothesis 4: The innovation of mature firms benefits more from the specialized innovative resources of network embedding depth, and this positive effect strengthens as firms increase in age.**

## Materials and Methods

### Data

This study uses listed company data from CSMAR and the public resume data from LinkedIn China for the time period from 2000 to 2015. We made several efforts to obtain an ideal research sample.

First, referring to Ge et al. (2016), we retrieved 165,299 online resumes with distributed web crawlers and obtained 376,378 personal employment records. We obtained a total of 184,877 employment records from China’s listed companies. Next, we filtered the data by retaining the personal employment records for top managers and R&D talent that were dated from 2000 to 2015. We deleted the records in which the job description indicated an internship or a default value that did not provide the company name or position name. Ultimately, we sorted 89,671 employment records from China’s listed companies. It is important to note that we identified individuals hired successively by different companies as flowing talent, and we considered their starting year with the subsequent company as the flowing year.

Relying on the method from Godart et al. (2014), we used the talent flow data to develop an interorganizational talent flow network. In the network, the nodes represent different firms and the connecting lines represent the flow of talent between firms. We checked only for the existence of talent flow and not the actual amount. Thereafter, we applied Pajek 3.0 to calculate the degree centrality and closeness centrality, which are used to measure the embedding breadth and depth of firms in the talent flow network.

Next, we collected indicators for the listed companies from China for the time period from 2000 to 2015. These indicators included the number of inventions and patents (*INNO*), firm age (*Age*), number of employees (*Size*), asset-liability ratio (*Lev*), revenue growth rate (*Growth*), return on assets (*ROA*), Tobin’s Q value (*Tobin’s Q*), ownership attributes (*SOE*), industry attributes (*Industry*), etc. We used the natural logarithm of the sum of *INNO* to represent the innovation performances of firms. And the natural logarithm of *Size* was used to control the effect of the size of the firms. Then, *Tobin’s Q* was introduced to control the effect of the firms’ future investment opportunities. *ROA* and *Growth* are applied to control the impact of the firms’ growth capacity, and *Lev* was included to control the impact of the financing structures for the firms.

Finally, by using the year and the listed company codes, we matched the talent flow network dataset with the CMSAR dataset and obtained an unbalanced panel dataset for the period of time from 2000 to 2015. The dataset included the names and ages of the firms, their embedding breadth and depth in the talent flow network, their innovation performances, and several control variables. Furthermore, we filtered the data by excluding financial firms, real estate companies, and firms with an asset-liability ratio greater than one. We also winsorized the continuous variables at the level of 1%. Ultimately, 3,027 valid samples were obtained for the main effect regression model because some samples only contained data for listed companies and some listed companies disclosed incomplete information.

### Measures

#### Network Embedding Breadth and Depth

Social network theory holds that, actors in the same network can obtain the other actors’ resources through the connections between them (Wang et al., 2017). Our study suggests that, the mobility of strategic talents between firms generates a talent flow network among them (Dokko and Rosenkopf, 2010; Godart et al., 2014). The focal firms in the network are able to absorb critical innovative resources including information, knowledge, and technologies from the other firms (Shipilov et al., 2017). When a firm is embedded more broadly in the network, it can get connected to more external organizations through talent flow, and thereby acquiring more abundant and diversified innovative resources. When a firm gets embedded more deeply in the network, it can establish stronger connections with the other firms, thus obtaining deeper tier and more pivotal resources.

We use degree centrality and closeness centrality to represent firms’ embedding breadth and depth in the talent flow network. Degree centrality measures the number of external organizations that the focal firm is directly connected with. Greater degree centrality means that the focal firm is directly connected to a larger number of external organizations. To some extent, it indicates the richness and diversity of the focal firm’s connections with the external organizations, thereby representing the focal firm’s embedding breadth in the network. Closeness centrality measures the distance between the focal firm and the other firms in the network. The greater the closeness centrality is, the shorter is the distance between the focal firm and those who are directly or indirectly connected to it. To a certain degree, it reflects how strong the focal firm is connected with the other firms, thereby representing the focal firm’s embedding depth in the network. Referring to Godart et al. (2014), this paper uses pajek 3.0 to build up the talent flow network that is shaped by inter-organizational talent mobility. Then, we use degree centrality and closeness centrality to measure firms’ embedding breadth and embedding depth in the network, respectively.

##### Network Embedding Breadth

Network Embedding Breadth (*Bre*) is represented by the standardized degree centrality. Equation (1) shows the calculations: *i* represents a specific firm that is involved in the talent flow network; *j* represents the other firms but *i* in the same year; *X*_*ji*_ stands for the connections between firms; *g* is the number of firms in the network in a specific year. The talent flow networks we build for different years are different in scale. Hence, the standardization of degree centrality is applied to eliminate the scale differences.


(1) $$B\,r\,e_{i}=\frac{\sum_{\text{j}}X_{\text{ji}}}{g-1}$$

##### Network Embedding Depth

Closeness centrality, which measures the distance between firm *i* and the other firms *j* in the network, represents firms embedding depth in the talent flow network. Here, *d*_*ij*_ is the distance between *i* and *j* in a certain year (the length of the shortcut between two nodes). Eq. (2) is the reciprocal of the distance between *i* and *j* in the same year.


(2) $$D\,e\,p_{i}={\left[\sum_{j=1}^{g}d_{i\,j}\right]}^{-1}$$

#### Firm Life Cycle

Firm life cycle has been defined with multiple factors including firm age, cash flow and the maturity of management method. Van Wissen (2002) states that defining firm life cycle by age is the most effective and reasonable way. Consulting the method of Huergo and Jaumandreu (2004) and Balasubramanian and Lee (2008), this paper uses firm age to define firm life cycle. We first calculate the age of sample firms at different years according to the date of establishment. Then, with the quantile of firm age, we obtain the age scales of firms. The values of firms’ age scales are restricted between 1 and 5, which represents five different stages of firms’ life cycle. But actually, firms’ life cycle can be divided into more different stages. Hence, to test the robustness of the regression results, we divided firms’ age scales into 3, 4, and 6 subsections, respectively.

#### Firms’ Innovative Performance

Firms’ innovative performances (*INNO*) usually refers to the presentation of new products and new ideas. Consulting the measurement method in Ahuja (2000), this paper adopts the logarithm of the sum of firms’ inventions and patents (inventions, utility models and product design) to represent firms’ innovative performances.

### Empirical Model

This paper uses the listed company panel data. We apply both OLS and fixed effects model in the main regression. In the OLS model, the fixed effects of year and industry attributes are controlled, and robustness estimators are used in the regression. Meanwhile, we tried to minimize the endogenous problems in the robustness test by lagging the explanatory variables, replacing the *INNO* measurement indicators and altering the defining standard of firm life cycle in the research models.

Referring to Laursen and Salter (2006) and Shipilov et al. (2017), this paper builds up an empirical model (Equation 3) to examine how network embedding breadth and depth are associated with firms’ innovations, and test the moderating effect of firm age on it. *INNO*_*it*_ is the innovation of firm *i* in year *t*. β is the coefficient. *Industry*_*i*_ and *Year*_*t*_ represent the fixed effects of industry and year. *ε_*it*_* is the random disturbance term.

Hypothesis 1 is that network embedding breadth has promoting effect on firms’ innovative performances. If it is true, β_1_, the coefficient on *Bre* in model (3) should be significantly positive. Likewise, Hypothesis 2 assumes that network embedding depth can enhance firms’ innovative performances. If it is supported, β_2_, the coefficient on *Dep* in model (3) should be significantly positive too. Hypothesis 3 supposes that network embedding breadth is more beneficial to young firms’ innovations, but the positive effects decline as firm age grows. If it is true, β_2_ to β_5_ in model (4) should all be significantly negative. Hypothesis 4 assumes that network embedding depth is more favorable to mature firms’ innovations, and this positive effect grow stronger with firm age increasing. If it is valid, β_7_ to β_10_ in model (4) should all be significantly positive.


(3) $$\begin{array}{cc} INNO_{it}= & \alpha_{0}+\beta_{1}Bre_{it}+\beta_{2}Dep_{it}+\beta_{3}ROA_{it}+\beta_{4}Growth_{it}\\ & +\beta_{5}Size_{it}+\beta_{6}Tobin\prime s\;Q_{it}+\beta_{7}Lev_{it}+\beta_{8}SOE_{it}\\ & +\beta_{9}Age_{it}+Industry_{i}+Year_{i}+\varepsilon_{it} \end{array}$$

(4) $$\begin{array}{cc} INNO_{it}= & {\text{α}}_{0}+{\text{β}}_{1}Bre_{it}+{\text{β}}_{2}AS_{2}*Bre_{it}+{\text{β}}_{3}AS_{3}*Bre_{it}+{\text{β}}_{4}AS_{4}\\ & *Bre_{it}+{\text{β}}_{5}AS_{5}*Bre_{it}+{\text{β}}_{6}Dep_{it}+{\text{β}}_{7}AS_{2}*Dep_{it}+{\text{β}}_{8}AS_{3}*Dep_{it}+{\text{β}}_{9}AS_{4}*Dep_{it}+{\text{β}}_{10}AS_{5}*Dep_{it}+{\text{β}}_{11}\\ & ROA_{it}+{\text{β}}_{12}Growth_{it}+{\text{β}}_{13}Size_{it}+{\text{β}}_{14}Tobin^{\prime }sQ_{it}+{\text{β}}_{15}Lev_{it}+{\text{β}}_{8}SOE_{it}+{\text{β}}_{16}Age_{it}+Industry_{i}+Year_{i}+\varepsilon_{it} \end{array}$$

## Results

### The Descriptive Statistics and Correlation Analyses

The descriptive statistics and the correlation analyses of the main variables are listed in Table 1.

**TABLE 1:** Means, standard deviations, and correlations.

	*Obs.*	*INNO*	*Bre*	*Dep*	*Age*	*ROA*	*Growth*	*Size*	*Tobin’s Q*	*Lev*
*INNO*	3,027	1								
*Bre*	3,027	0.259***	1							
*Dep*	3,027	0.247***	0.307***	1						
*Age*	3,027	0.045**	0.0120	−0.032*	1					
*ROA*	3,027	0.054***	0.0230	0.109***	−0.082***	1				
*Growth*	3,027	–0.009	0.052***	0.0290	−0.033*	0.309***	1			
*Size*	3,027	0.475***	0.169***	0.238***	0.120***	–0.0300	−0.050***	1		
*Tobin’s Q*	3,027	−0.201***	−0.042**	0.054***	−0.181***	0.452***	0.163***	−0.397***	1	
*Lev*	3,027	0.213***	0.099***	0.045**	0.203***	−0.397***	–0.00500	0.496***	−0.501***	1
Mean	–	2.924	0.041	0.005	19.04	0.051	0.182	22.18	2.194	0.428
Std.	–	1.461	0.234	0.012	4.533	0.054	0.300	1.321	1.858	0.189
Min	–	0.693	0	0	6	–0.140	–0.448	19.99	0.200	0.053
Max	–	8.752	5.006	0.053	38	0.209	1.528	26.33	10.17	0.821

***p < 0.01, **p < 0.05, *p < 0.1.

### Regression Results

Table 2 shows the results of regressions between firms’ network embedding features, firm life cycle and firms’ innovative performance. The impacts of network embedding breadth and depth on firms’ innovations are tested. So are the impacts at different life stages. That is, all Hypotheses 1, 2, 3, and 4 are tested in the regressions.

**TABLE 2:** Talent inflow network, life cycle, and firms’ innovation.

	Column (1)	Column (2)	Column (3)	Column (4)
	OLS	FE	OLS	FE
*Bre*	0.853***	0.381***	2.929***	2.979**
	(0.075)	(0.149)	(0.551)	(1.248)
*Age2_Bre*			−0.763	−2.640**
			(1.155)	(1.295)
*Age3_Bre*			−2.144***	−2.639**
			(0.558)	(1.258)
*Age4_Bre*			−2.076***	−4.228***
			(1.048)	(1.446)
*Age5_Bre*			−2.508***	−2.980**
			(0.704)	(1.489)
*Dep*	8.106***	3.228*	−0.399	2.286
	(2.045)	(1.658)	(4.420)	(4.208)
*Age2_Dep*			5.136	−5.410
			(5.607)	(5.462)
*Age3_Dep*			12.372**	5.061
			(6.429)	(5.539)
*Age4_Dep*			15.775**	10.193*
			(6.672)	(5.842)
*Age5_Dep*			4.577	−4.057
			(6.367)	(5.648)
*ROA*	1.848***	0.099	1.773***	1.871***
	(0.520)	(0.522)	(0.522)	(0.539)
*Growth*	−0.139*	−0.062	−0.135*	−0.026
	(0.080)	(0.066)	(0.080)	(0.068)
*Size*	0.518***	0.525***	0.515***	0.329***
	(0.029)	(0.052)	(0.029)	(0.048)
*Tobin’s Q*	0.026*	−0.024	0.023	−0.116***
	(0.016)	(0.018)	(0.016)	(0.015)
*Lev*	0.041	−0.302	0.015	−0.159
	(0.150)	(0.212)	(0.151)	(0.222)
*Age*	0.006	−	0.004	−
	(0.005)	−	(0.005)	−
*SOE, Industry, Year*	Control	Control	Control	Control
Obs.	3,027	3,027	3,027	3,027
*R* ^2^	0.439	0.321	0.440	0.222
*F*	32.11***	46.07***	29.31***	34.08***

Standard errors are in parentheses. ***p < 0.01, **p < 0.05, *p < 0.1.

As shown in Table 2: Model 1 is the OLS regression of *Bre* (firms’ network embedding breadth) and *Dep* (firms’ network embedding depth) to *INNO* (firms’ innovative performances), with a number of variables controlled; Model 2 is the fixed effects regression of *Bre* and *Dep* to *INNO*; in Model 3, two interaction terms are added to the OLS regression in Model 1, namely, the interaction term of *Bre* and firm age (*Age2_Bre*, *Age3_Bre*, *Age4_Bre*, *Age5_Bre*), and the interaction term of *Dep* and firm age (*Age2_Dep, Age3_Dep, Age4_Dep, Age5_Dep*); Model 4 is the fixed effects regression of all the variables from Model 3.

The regression results of Model 1 present significant positive impact of *Bre* on *INNO* (*b* = 0.853, *p* < 0.01). That is, greater network embedding breadth enhances firms’ innovative performance, which is consistent with Hypothesis 1. Model 1 also reports significant positive influence of *Dep* on *INNO* (*b* = 8.106, *p* < 0.01), meaning that greater network embedding depth promotes firms’ innovative performances, supporting Hypothesis 2. In addition, the results of the fixed effects regression in Model 2 show significant positive effects of both *Bre* and *Dep* on *INNO* (*b* = 0.381, *p* < 0.01; *b* = 3.228, *p* < 0.1), which further supports Hypotheses 1 and 2.

Then, the regression results of Model 3 show that: (i) The regression coefficient of *Bre* on *INNO* (*b* = 2.929, *p* < 0.01) is larger than that of *Dep* on *INNO* (*b* = -0.339, *p* > 0.1); (ii) With firm age increasing, the coefficients on the interaction terms of *Bre* and firm age (*Age2_Bre* to *Age5_Bre*) show a significant decreasing trend, while on the contrary, the coefficients on the interaction terms of *Dep* and firm age increase gradually. The results are consistent with Hypotheses 3 and 4.

Meanwhile, the fixed effects regression in Model 4 have similar results: (i) The regression coefficient of *Bre* on *INNO* (*b* = 2.979, *p* < 0.05) is larger than that of *Dep* on *INNO* (*b* = 2.286, *p* > 0.1); (ii) The coefficient on the interaction term *Age2_Bre* is -2.640 (*p* < 0.05), indicating that when network embedding breadth increases by 1 unit, the innovations of firms at Age 2 is reduced by 2.64% compared to that at Age 1; (iii) The coefficients on *Age3_Bre*, *Age4_Bre* and *Age5_Bre* are -2.639 (*p* < 0.05), -4.228 (*p* < 0.01), and -2.980 (*p* < 0.05), respectively, implying that the influence of *Bre* on the innovation of firms at Age 4 is minimal. But after Age 4, the impact grows stronger; (iv) The coefficients on *Age2_Dep*, *Age3_Dep*, *Age4_Dep* and *Age5_Dep* are -5.410 (*p* > 0.1), 5.061 (*p* > 0.1), 10.193 (*p* < 0.1), and -4.057 (*p* > 0.1), respectively, showing that the impact of *Dep* on the innovation of firms at Age 4 is maximal. But the influence of *Dep* on the innovation of firms at *Age 2*, *Age3* and *Age 5* are not significantly different from that at *Age 1*. Apparently, the regression results support Hypotheses 3 and 4 again.

**FIGURE 1:**
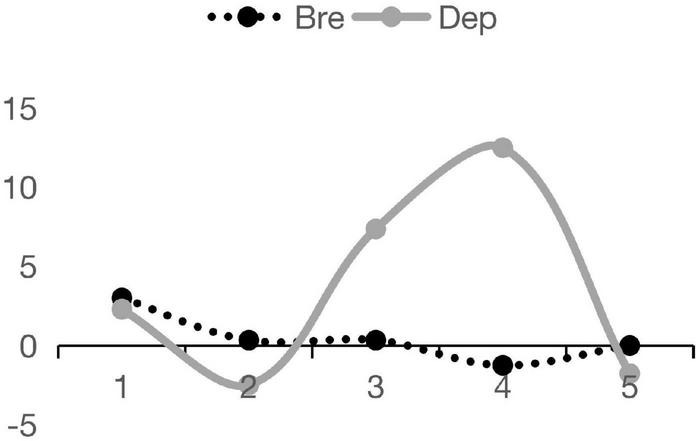
Network embedding breadth, depth and firms’ innovation performances.

Based on Model 4, Figure 1 illustrates how strong the impacts of *Bre* and *Dep* on *INNO* can be at different life stages of firms: (1) For firms at *Age 1*, network embedding breadth seems to have stronger impact on firms’ innovative performances than the depth. But when it comes to the stage of *Age 3*, the influence of network embedding depth on firms’ innovations excels that of the breadth, and henceforth plays the leading role; (2) The impact of *Bre* on *INNO* is U shaped, which means that it declines first as firm age increases, and then grows stronger. It reaches the minimum value at the stage of *Age 4*; (3) With firm age growing, the impact of *Dep* on *INNO* demonstrates a wavy trend. It declines first, and goes up next, and then decreases again. It reaches the peak at the stage of *Age 4*. To conclude, the association between network embedding features and firms’ innovations is not just some simple linear relation. Actually, the association between *Bre* and *INNO* is slightly U shaped, while that between *Dep* and *INNO* is U shaped first and then becomes inverted-U shaped.

### Robustness Test

There might be a few endogenous problems in this research: (1) simultaneity bias; (2) The lag problem of *INNO;* (3) The measurement of *INNO;* (4) The standard of firm age divisions. To address these problems, a series of robustness tests were conducted (see Tables 3, 4 for details).

**TABLE 3:** Robustness test 1.

	OLS	FE	FE
	Column (1)	Column (2)	Column (3)
*Bre*_*t*–1_	0.773***	0.492***	0.443*
	(0.098)	(0.173)	(0.239)
*Dep*_*t*–1_	7.951***	4.144*	1.597*
	(2.596)	(2.287)	(0.956)
*Controls*	YES	YES	YES
Obs.	1,747	1,747	527
*R* ^2^	0.503	0.213	0.847
*F*	26.59***	36.28***	95.98***

Standard errors are in parentheses. ***p < 0.01, *p < 0.1; Controls represents variables including ROA, Growth, Size, Tobin’s Q, Lev, Age, SOE, Industry, Year.

**TABLE 4:** Robustness test 2.

	Column (1)	Column (2)	Column (3)
	Three categories	Four categories	Six categories
Bre	2.643***	2.755***	3.113***
	(0.538)	(0.580)	(0.675)
*Age2_Bre*	−1.848***	1.176	−0.368
	(0.544)	(0.909)	(0.993)
*Age3_Bre*	−2.243***	−1.973***	1.806
	(0.705)	(0.584)	(1.274)
*Age4_Bre*		−2.255***	−2.352***
		(0.716)	(0.680)
*Age5_Bre*			−2.427**
			(1.147)
*Age6_Bre*			−2.857***
			(0.812)
*Dep*	−0.287	1.305	−1.768
	(3.270)	(3.684)	(5.248)
*Age2_Dep*	12.819***	3.008	4.449
	(4.550)	(5.083)	(6.284)
*Age3_Dep*	9.829**	10.209*	4.773
	(4.907)	(5.477)	(6.817)
*Age4_Dep*		7.414	15.024**
		(5.589)	(7.211)
*Age5_Dep*			17.011**
			(7.381)
*Age6_Dep*			5.967
			(7.427)
*Controls*	YES	YES	YES
Obs.	3,027	3,027	3,027
*R* ^2^	0.440	0.441	0.443
*F*	30.77***	30.12***	28.93***

Standard errors are in parentheses. ***p < 0.01, **p < 0.05, *p < 0.1.

First, for the problems of simultaneity bias and the lag problem of *INNO*, we re−examined the main regression model (Model 1 and Model 2 in Table 3) by using the lagged values of *Bre* and *Dep* (*Bre*_*t*–1_ and *Dep*_*t*–1_). Theoretically, the enhanced innovation performance of this year does not influence the talent acquisition of the last year. And technically, this also examined the lagging problem of *INNO*. As can be seen in Table 3, the regression results of Model 1 and 2 still support Hypotheses 1 and 2.

Next, to address the measurement deviation of *INNO*, we used firms’ main business income to represent their innovative performances. We chose the sample firms from two industries, namely, software and information technology service industry and the manufacturing industry for computers, communication devices and other electronic equipment, which are characterized with high speed in product innovation and updates. In these firms, the main business income can be regarded as the production value of innovative products, which is usually used to measure firms’ innovative performances. As shown in Table 3, the regression results of Model 3 are still consistent with Hypotheses 1 and 2.

At last, to tackle the problem in firm age divisions, we re-examined the influence of *Bre* and *Dep* on firms at different stages of their life course, by adopting three age division methods, namely, 3, 4, and 6 subsections. The regression results in Table 4 show that: (1) No matter which age division method we apply, the regression coefficient on *Bre* is greater than that on *Dep*; (2) The trend of the interaction coefficient of network embedding features and firm age is still robust; (3) The coefficient on the interaction term of *Bre* and firm age is significantly negative, and gradually decreases with firm age growing; (4) The coefficient on the interaction term of *Dep* and firm age is significantly positive. It grows gradually with firm age increasing, and reaches the peak at higher age levels.

## Discussion

This paper examines how the embedding breadth and depth of firms in the talent flow network influence their innovation performances and how the effects change at different development stages. The results indicate the following: (1) greater network embedding breadth and depth enhance the innovation performances of firms; (2) during the early stages of the life cycle for firms, innovations benefit more from the diversified resources of the network embedding breadth, but this positive effect declines as firms increase in age; and (3) for mature firms, innovations benefit more from the deeper-level resources of the network embedding depth, and this positive effect strengthens as firms increase in age.

This paper primarily has two theoretical contributions. First, this study systematically researched the influence of network embedding breadth and depth on firms’ innovation performances by examining different network embedding features. Many previous studies examined the influence of network embedding breadth on firms’ innovations from the perspective of degree centrality (Godart et al., 2014; Shipilov et al., 2017) and found that firms that are embedded more broadly in the talent flow network achieve better innovation performances. In fact, when firms become broadly embedded in the network, they must occupy central positions to access more diversified information, knowledge, technology, and human capital. However, when considering resources and capability, not all companies can occupy central positions. Furthermore, not all companies need the abundant and diversified resources of the network to enhance their innovations. Thus, our study introduces the concepts of the breadth and depth of the external resource search (Laursen and Salter, 2006) into the talent flow network and suggests that embedding deeply in the talent flow network is another effective way for firms to access important and extensive resources. The regression results indicate that both network embedding breadth and depth have significant positive effects on firms’ innovation performances.

Additionally, this study considers the firm life cycle and discusses the impact of network embedding breadth and depth on the innovation performances of firms at different development stages. Thus, this study enriches the previous research regarding the relationship between network embedding features and firms’ innovation performances. The existing empirical studies have mainly examined the connection between network embedding breadth and the innovations of firms. Some studies have determined that this relationship is linear, but other studies have indicated the opposite view. The reason for this inconsistency may be that researchers have ignored the different characteristics of firms at different stages in their development. Hence, considering the life cycle of firms, we propose that different network embedding strategies enhance firms’ innovations at different stages.

Our results show that the impact of network embedding breadth has a U-shaped impact on the innovation performances of firms as they increase in age. The impact of network embedding depth is represented by a wavy line as it is first U-shaped and then becomes an inverted U. In other words, for firms in the early stages of the life cycle, their innovations rely more on the diversified innovative resources from the network embedding breadth, but the innovations of mature firms mainly rely on the pivotal resources from the network embedding depth.

To some extent, two principles explain the results of this study—“the best within the expertise” and “the best out of the expertise.” These principles are presented by the 3D creativity-making framework (Vuong and Napier, 2014, p. 304; Vuong et al., 2022), which explains that the key for innovation is “out of discipline” thinking, “within discipline” expertise, and a “disciplined process” (Napier and Nilsson, 2008). “Out of discipline” thinking means that firms exceed their own professional boundaries and audaciously integrate the acquired resources to seek more ideas that are potentially innovative. “Within discipline” expertise is when firms depend on their existing talent and knowledge to consider and screen the acquired resources to obtain useful insights and practical solutions. These two principles may coexist in the innovation process, but one may dominate the other as the innovation objectives change as firms increase in age.

Specifically, younger firms need more creative products to attract customers when they enter a particular product market (Klette and Skrotum, 2004; Coad et al., 2016). In other words, younger firms need more out-of-discipline thinking to generate more creative ideas and innovative outcomes. This requires younger firms to obtain more abundant and diversified resources. Hence, firms at the early development stages need to embed more broadly in the talent flow network. On the other hand, mature firms already have established products in the market. They have set their market position, and they have their own R&D professionals. The optimal strategy for mature firms is to refine their existing products rather than develop brand new products (Coad et al., 2016; Akcigit and Kerr, 2018). Therefore, they should focus more on within-discipline expertise to acquire more technical resources and develop practical solutions to improve their existing innovative products. Thus, mature firms should become more deeply embedded in the talent flow network and obtain more technical resources so their internal talent can improve their existing products.

Our study has two implications for the innovation management of firms. First, deliberate and purposeful talent flow management can effectively improve firms’ innovative performances. Our study found that both network embedding breadth and depth have significant positive effects on firms’ innovative performances. Stated differently, by purposefully managing the talent flow, the resource spillover effects of the network provide various resources, including knowledge, information, technology, and human capital, that benefit the innovations of firms. On the one hand, firms can deliberately maintain diversified sources of talent during the recruiting process to increase their network embedding breadth, which in turn brings more diversified resources. On the other hand, firms can acquire key talent from important nodes in the network and increase their network embedding depth. Subsequently, firms can acquire deeper-level resources, such as key talent from other leading innovative companies.

Second, firms at different ages should consider enterprise characteristics when embedding themselves in the talent flow network to maximize their innovative performances. Our research found that younger firms benefit more from the diversified resources of network embedding breadth, but more mature firms benefit from the deeper-level resources of network embedding depth.

In conclusion, firms should adjust their network embedding strategies according to their development stages. During the early stages of development, the primary strategic objective for firms is to occupy a position in the market and gain some market share rather than maximize their profits (Coad et al., 2016; Akcigit and Kerr, 2018). Accordingly, younger firms should increase their R&D expenditure and conduct a broad search for talent. By increasing their embedding breadth in the talent flow network, these younger firms can obtain various innovative resources that they can use to develop more creative products, attract more customers, and ultimately gain a position in the product market. Thus, younger firms must invest more in R&D and improve their embedding breadth in the talent flow network, even though they only receive limited income from the product market they have recently entered. This is the only way that younger firms can design more innovative products and gain market share. Controlling R&D input and seeking R&D outcomes are usually contradictory activities. Younger firms are generally trying to catch up during a time that enhancing R&D is essential. Inevitably, the R&D costs will reduce the profit level at this stage, but it will help firms gain a position in the market and surpass their competitors in the future.

The situation for mature firms is quite different. Their strategic objective is to maintain their current market share and simultaneously maximize their profits (Coad et al., 2016; Akcigit and Kerr, 2018). Thus, mature firms usually reduce their R&D input and control their R&D costs. Meanwhile, these firms should focus on increasing their embedding depth in the talent flow network to identify technical talent who can help optimize existing products. This allows mature firms to acquire more professional resources to improve their existing innovative products and to achieve the objectives of maintaining their market share and maximizing their profits. Thus, mature firms need to control their R&D costs, search for technical talent in a targeted way to improve their network embedding depth, and meet the objectives of product improvement and profit optimization.

## Data Availability Statement

Publicly available datasets were analyzed in this study. This data can be found here: https://www.gtarsc.com.

## Author Contributions

BS and BP contributed to the conception and design of the work, drafted the work, and revised it critically for important intellectual content. AR and BS contributed to the acquisition, analysis, and interpretation of data for the work and were also responsible for the approval of the version to be published. WL contributed to the analysis and interpretation of data for the work. All authors made substantial contributions to the article and approved the submitted version.

## Conflict of Interest

The authors declare that the research was conducted in the absence of any commercial or financial relationships that could be construed as a potential conflict of interest.

## Publisher’s Note

All claims expressed in this article are solely those of the authors and do not necessarily represent those of their affiliated organizations, or those of the publisher, the editors and the reviewers. Any product that may be evaluated in this article, or claim that may be made by its manufacturer, is not guaranteed or endorsed by the publisher.
